# Osteogenic Differentiation in Healthy and Pathological Conditions

**DOI:** 10.3390/ijms18010041

**Published:** 2016-12-27

**Authors:** Maria Teresa Valenti, Luca Dalle Carbonare, Monica Mottes

**Affiliations:** 1 Department of Medicine, Section of Internal Medicine D, University of Verona, 37128 Verona, Italy; luca.dallecarbonare@univr.it; 2 Department of Neurosciences, Biomedicine and Movement Sciences, Section of Biology and Genetics, University of Verona, 37134 Verona, Italy; monica.mottes@univr.it

**Keywords:** bone, osteoblasts, mesenchymal stem cells, remodeling, Runt-related transcription factor 2 (*RUNX2*), Wingless-Type MMTV Integration Site Family (WNT), Pigment Epithelium Derived Factor (PEDF), microRNAs, osteopenia

## Abstract

This review focuses on the osteogenic differentiation of mesenchymal stem cells (MSC), bone formation and turn-over in good and ill skeletal fates. The interacting molecular pathways which control bone remodeling in physiological conditions during a lifelong process are described. Then, alterations of the molecular pathways regulating osteogenesis are addressed. In the aging process, as well as in glucocorticoid-induced osteoporosis, bone loss is caused not only by an unbalanced bone resorption activity, but also by an impairment of MSCs’ commitment towards the osteogenic lineage, in favour of adipogenesis. Mutations affecting the expression of key genes involved in the control of bone development occur in several heritable bone disorders. A few examples are described in order to illustrate the pathological consequences of perturbation in different steps of osteogenic commitment, osteoblast maturation, and matrix mineralization, respectively. The involvement of abnormal MSC differentiation in cancer is then discussed. Finally, a brief overview of clinical applications of MSCs in bone regeneration and repair is presented.

## 1. Introduction

Bone is a dynamic organ, able to replace old or disrupted tissue through a remodeling process. This process is indispensable for bone to adjust to the continuous mechanical changes required by skeletal functions in varying environmental conditions. Osteoblasts and osteoclasts are the pivotal cells involved in bone turnover: they are responsible for bone formation and bone resorption, respectively. In particular, osteoblasts arise from the osteogenic differentiation of mesenchymal stem cells through a process regulated in different steps. Osteocytes, other important skeletal cells that derive from mature osteoblasts and are surrounded by secreted extracellular matrix, regulate osteoblast and osteoclast activity and, consequently, maintain bone homeostasis. Alterations of mesenchymal stem cells may occur during commitment or differentiation towards the osteogenic lineage, causing demineralization or bone loss in different pathological settings.

## 2. Bone Remodeling

**Figure 1:**
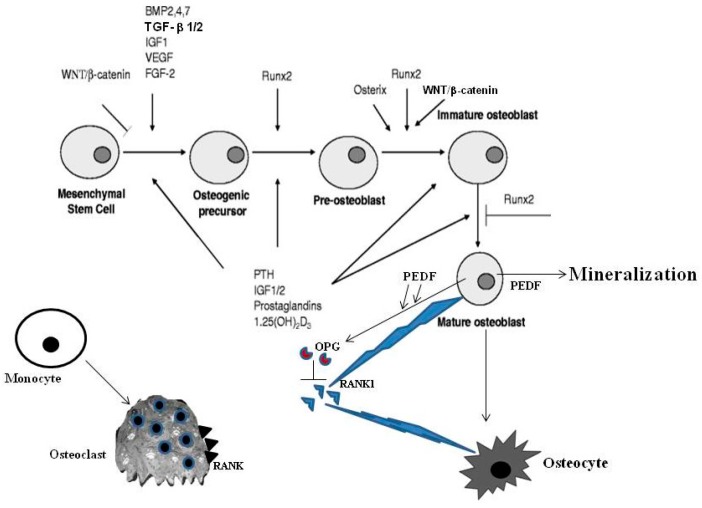
Schematic representation of cell types and major regulators of the molecular pathways involved in osteoblastogenesis and bone formation.

Bone is the major supportive tissue of the body; it protects vital organs and provides storage for calcium and phosphate. Cortical and trabecular bone, in particular, are the two compartments designated mainly for mechanical and metabolic functions, respectively. Two major modes of bone formation can be distinguished: intramembranous ossification and endochondral ossification. The first process (direct ossification), which occurs in the flat bones of the neuro- and viscerocranium and in part of the clavicle [1], is characterized by the condensation of mesenchymal stem cells which, after the commitment to osteo-progenitors, become osteoblasts. Mature osteoblasts can become bone lining cells or evolve to osteocytes or may undergo apoptosis. Osteocytes may behave as mechanical sensors by acting on organic and inorganic molecules, under mechanical stimuli; in this way they are able to remodel the perilacunar environment and contribute to maintaining bone function [2]. Conversely, during endochondral ossification (indirect ossification), which occurs in long bones, vertebrae and in the skull base and the posterior part of the skull [1], the mesenchymal stem cells differentiate into cartilage first, and this cartilage is later replaced by bone, increasing the ability to counteract the compression [3]. Modeling, occurring during the growth process, as well as remodeling, a lifelong process, are characterized by bone turnover. Bone remodeling takes place under the control of various players, i.e., parathyroid hormone (PTH), calcitonin, vitamin D, growth hormone (GH), steroids, soluble cytokines and growth factors (i.e., Macrophage Colony-Stimulating Factor (M-CSF), Receptor Activator of Nuclear κB Ligand (RANKL), Vascular Endothelial Growth Factor (VEGF), Interleukin-6 (IL-6) family). Different stimuli, such as micro-fractures or factors related to bone microenvironment, induce osteoblasts to produce RANKL which, in turn, interacts with its receptor RANK (Receptor Activator of Nuclear κB), expressed by osteoclasts. This interaction determines osteoclast polarization with the generation of the ruffled border, a site of the secretion of enzymes required for bone resorption. Osteoblasts, which interact with bone matrix through integrins (e.g., α1β1, α2β1 and α5β1), synthesize type I collagen (that represents 90% of the proteins in the bone matrix). In addition, Procollagen I N-terminal peptide (PINP) is produced by amino-terminal and carboxy-terminal splicing of type I procollagen in osteoblasts and it is considered a marker of bone formation [4]. Unmineralized osteoid is composed of type I collagen together with other fibrillar collagens, bone proteins (osteopontin, bone sialoprotein and osteocalcin), proteoglycans, fibronectin, glycosaminoglycans, etc. PEDF (pigment epithelium derived factor) is necessary for osteoblast development; it stimulates the expression of osteogenic genes and mineral deposition [5] and it also stimulates the production of osteoprotegerin, thus inhibiting osteoclast maturation. PEDF knock-out mice show bone defects and a propensity for fractures, and represent a good model for the human disease osteogenesis imperfecta type VI (see Section 4) [6]. Mineralization then occurs thanks to the activity of osteoblast phosphatases, releasing phosphates that together with calcium form hydroxyapatite crystals [Ca_10_(PO_4_)_6_(OH)_2_] [7]. Some osteoblasts get imprisoned into the mineralized matrix and after a morphologic change, acquire a stellar shape with many extensions. These cells, called osteocytes, form a network and are able to produce signaling through bone tissue (Figure 1).

## 3. Molecular Pathways

Bone morphogenetic proteins (BMP) [8] and WNT [9] are important signaling pathways for osteogenesis. The BMP pathway starts by activating SMAD intracellular proteins which, in turn, control the expression of the master gene *RUNX2* (Runt-related transcription factor 2). RUNX2 is the transcription factor that induces the commitment of mesenchymal stem cells to osteogenic lineage and acts upstream from the other osteoblast-specific transcription factor OSTERIX and other specific osteoblastic genes such as *SPARC* (osteonectin), *SPP1* (osteopontin), and *COL1A1* (type I collagen). RUNX2 expression is also regulated by the WNT pathway which plays an important role in bone formation. WNT proteins are involved in many biological processes such as organogenesis, tissue regeneration and tumorigenesis. The canonical (or classic) WNT pathway is represented by WNT/β-catenin signaling. The canonical WNT pathway acts by either inhibiting or inducing osteoblast formation depending on the level of differentiation of progenitor cells and it controls bone resorption by increasing the osteoprotegerin (OPG)/RANKL ratio [10]. In fact, OPG and RANKL are produced by osteoblasts that activate (by RANKL) or inhibit (by OPG, the decoy RANKL receptor) osteoclasts [11] A schematic representation of this complex network of signaling pathways involved in osteogenesis is shown in Figure 1. The non-canonical WNT signaling pathway also regulates osteoblast differentiation, since it inhibits the expression of PPARγ, the adipogenic transcription factor [12].

Furthermore, in addition to the BMP and WNT pathways, systemic hormones, such as parathyroid hormone (PTH), glucocorticoids, estrogens, and local growth factors such as bone transforming growth factor-β (TGF-β1/2), insulin-like growth factor (IGF), fibroblast growth factor 2 (FGF-2), vascular endothelial growth factor (VEGF), cytokine modulators (prostaglandins) and MAPK (Mitogen-activated protein kinases) signaling, regulate osteogenic commitment or differentiation of mesenchymal cells [13]. Recently, it has been shown that PIN1 (Peptidyl-prolyl *cis-trans* isomerase Never in Mitosis gene A (NIMA)-interacting 1) interacts with RUNX2, SMAD1/5, and β-catenin and it is involved in osteoclastogenesis, suggesting that this enzyme plays an important role in bone regulation [14].

Besides the pathways described above, epigenetic factors, such as DNA methylation, microRNA (miRNA), and chromatin structure modification, regulate osteogenesis [15]. In particular, miRNAs, short, non-coding RNAs, may affect both osteoblast lineage/bone formation and osteoclast lineage/bone resorption [16]. Post-transcriptional regulation of osteoblastogenesis by miRNAs may affect the expression of RUNX2 (e.g., miR-34c, miR-133a, miR-135a, miR-137, miR-205, miR-217, miR-338, miR-23a, miR-30c, miR-204/211, miR-103a) and Osterix (OSX) (e.g., miR-31, miR-93, miR-143, miR-145, miR-637, miR-214). The expression of type I collagen genes may also be affected by miRNAs (e.g., miR-29, miR-Let7) [16]. Even if the involvement of miRNAs in osteoclastogenesis has been poorly investigated so far, it has been reported that miR-155, miR-223, miR-124, miR-21 miR-29, and miR-503 may affect osteoclast differentiation and maturation by direct or indirect inhibition/upregulation [17,18].

Exosomes, small vesicles released by different cells including osteoblasts, contain various molecules such as proteins and RNA. Among the RNAs, exosomes contain mRNAs and miRNAs [19]. Recently, it was demonstrated that mineralizing osteoblasts produce exosomes containing different miRNAs that affect mesenchymal stem cells by enhancing osteogenic differentiation [20]. The authors reported that this effect may be due to the upregulation of β-catenin expression by miRNAs. Therefore, this finding highlights the crosstalk and the positive feedback between mature osteoblasts and mesenchymal stem cells.

## 4. MSC and Systemic Disorders Affecting Bone

As described above, the osteogenic differentiation of committed mesenchymal stem cells is controlled by various extracellular signals. Therefore, alterations of molecular pathways regulating osteogenesis may cause bone damage. MSCs can differentiate either into adipocytes or osteoblasts and the balance between these two lineages is important for bone health. Unfortunately, this balance may be altered by various factors. Bone loss can be due to different pathogenetic mechanisms, primarily to an increased resorption caused by the increased activity and number of osteoclasts. In such pathological conditions, an increased marrow adiposity has been demonstrated [21]. This finding has been explained as a consequence of an unbalanced MSCs commitment in favor of adipogenesis. We have shown that, in osteoporotic patients, ox-PAPCs (modified lipoproteins derived from the oxidation of arachidonate-containing phospholipids) affect osteogenesis by enhancing the adipogenesis of MCSs [22]. Bone loss and increased bone marrow adiposity have been shown also in glucocorticoid-induced osteoporosis (GIOP), a common form of secondary osteoporosis, consequent to the imbalance between osteogenesis and adipogenesis due to glucocorticoid treatment [23]. In the aging process, an alteration of MSC commitment involving a decrease in proliferation and osteogenic differentiation with enhanced adipogenesis has been observed [24]. Consequently, aging-associated bone loss is not caused exclusively by increased osteoclastic activity, but also by an impairment of MSC commitment toward the osteogenic lineage. Recently, we showed that hypoxia promotes sickle cell bone disease in a murine model by affecting osteogenesis and, in particular, downregulating *Runx2* gene expression [25]. Osteopenia is found also in chronic kidney diseases, where different bone loss patterns can be identified, characterized either by increased bone turnover and osteoclastic activity or by decreased turnover associated with decreased osteoblast differentiation [26]. All these findings underline the pivotal role of MSCs and their differentiation to osteogenic lineage in the pathogenesis of bone diseases. Disturbance of MSC differentiation is an interesting field of study and MSCs represent important targets for skeletal regenerative therapy.

## 5. MSCs and Heritable Bone Diseases

Perturbation/disruption of the complex molecular pathways controlling MSC proliferation and osteogenic commitment may be determined by mutations affecting key genes in bone development. A few paradigmatic heritable bone disorders consequent to dysfunctions in different steps of the osteogenic pathway are discussed below.

Fibrodysplasia ossificans progressiva (FOP, OMIM #135100) is a rare, disabling autosomal dominant disorder, characterized by intermittently heterotopic endochondral bone formation and malformed big toes. Most cases are sporadic, due to a recurrent de novo point mutation (c.617G>A) in the *ACVR1* gene, which codes for the activin A type 1 receptor. The consequent single amino acid substitution (R206H) elicits a gain-of-function effect in the mutant receptor, increasing its sensitivity to BMPs. In vitro disease models, i.e., induced pluripotent stem cells (iPSC) derived from FOP patient fibroblasts, have shown that the ACVR1 R206H mutation confers an increased tendency towards cartilage formation and mineralization, with a transient increase in the expression of osteogenic markers [27]. Interestingly, recent experiments in the cellular FOP model demonstrated that significant downregulation of the aberrant BMP signaling and inhibition of chondro-osseous differentiation were achieved by treating cells with a soluble recombinant fusion protein, ACVR1-Fc, containing the extracellular domain of wild-type ACVR1 and the Fc portion of human IgG1 [28].

Cleidocranial dysplasia (CCD, OMIM #119600) is a rare autosomal dominant heritable disorder characterized by absent/hypoplastic clavicles, persistently open/delayed closure of fontanels, worminan bones, abnormal dentition, and other skeletal changes. Various mutations (mostly causing loss of function) of RUNX2, the master gene for MSC osteogenic commitment, have been identified as the cause of CCD. It has been shown that Runx2-null mice do not form bone and die just after birth, while heterozygous Runx2-deficient mice (^+/−^) exhibit CCD features and reduced alkaline phosphatase activity [29]. MSCs of a CCD patient heterozygous for a RUNX2 mutation resulting in a truncated protein (^+/m^) showed a reduced proliferative potential and a reduced ability to differentiate into osteoblasts [30].

Osteogenesis imperfecta (OI) is a clinically heterogeneous disorder characterized by fragile, deformed bones and osteopenia. For many years it has been considered a collagen disorder. More than 85% of OI cases are actually due to dominant mutations in either of the two genes encoding type I collagen α chains (COL1A1 and COL1A2). Both quantitative and qualitative defects in the most abundant protein in the bone matrix may produce different clinical types. In recent times, thanks to exome-wide studies, new disease genes have been identified, concurring with the definition of the broad OI phenotype. Other rare severe/lethal OI types which show a recessive inheritance (e.g., OI type VII, OMIM# 610682; OI type VIII OMIM#610915) are also somehow collagen-related. Homozygous or compound heterozygous mutations in genes coding for collagen-modifying enzymes produce type I collagen trimers which are incorrectly folded or post-translationally overmodified. The consequent impairment in collagen secretion and extracellular matrix deposition jeopardizes correct bone mineralization [31]. Recent molecular findings of causative mutations for rare recessive forms of OI have clarified specific defects in the osteogenic commitment of progenitors. In particular, autosomal recessive-type VI OI (OMIM #613982) has peculiar histological features, revealing defects in the mineralization process, first described by Glorieux and colleagues [32]. Patients with type VI OI have homozygous or compound heterozygous null mutations in SERPINF1, the gene coding for PEDF.

**Figure 2:**
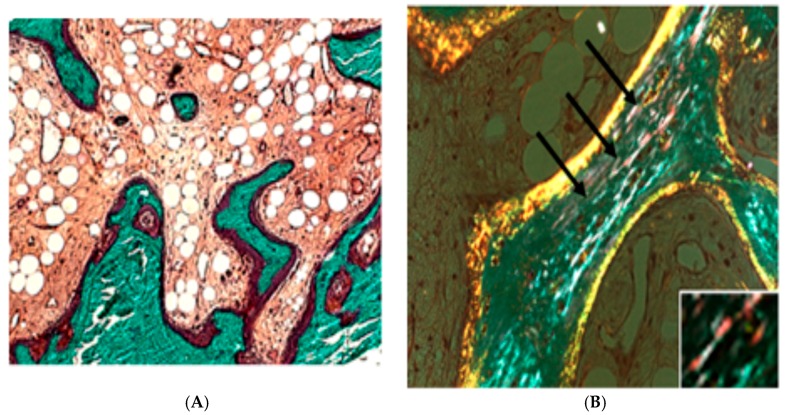
Defects in bone mineralization due to the lack of PEDF. (**A**) Iliac bone section of a patient affected by OI type VI. A large amount of unmineralized osteoid (in red) and resorption lacunae are visible. (**B**) Bone section under polarized light. Black arrows point to the so called “fish-scale” pattern (magnification 200×) (Reproduced from *J. Bone Miner. Res.* 2012, *50*, 343–349, with permission of the American Society for Bone and Mineral Research). The insert in (**B**) shows an enlargement of fish scale (2000×).

Children with type VI OI appear normal at birth, but they start fracturing within eight to 12 months of age and then develop a severe progressively deforming bone dysplasia, leading to the loss of autonomous walking. The bone histology of type VI patients is peculiar: it reveals increased amounts of unmineralized osteoid and anomalies in the orientation of lamellae (Figure 2).

Other recessive OI forms have been associated with mutations in two key genes controlling osteoblast differentiation: SP7/Osterix (OI type XII OMIM#613849) and WNT1 (OI type XV, OMIM#615220) [31,32].

Studies on the rare conditions described above contributed further knowledge about the major actors in bone development and mineralization.

Hypophosphatasia (HPP, OMIM#241500) is a genetic condition associated with mutations in Alkaline Phosphatase Liver/bone/kidney (*ALPL*) gene which encodes the tissue-non-specific alkaline phosphatase isozyme (TNSALP) [33]. Different mutations in ALPL may lead to the production of defective/inactive TNSALP. As a consequence, the concentration of inorganic pyrophosphate (PPi) increases in the bone matrix, thus impairing bone mineralization and disturbing calcium and PPi homeostasis. The pathogenetic mechanisms leading to bone hypomineralization in HPP have been examined fairly well; biomedical research directed toward treatment has focused mainly on enzyme replacement therapy. Results have been unsatisfactory, nevertheless. For the future, more innovative therapeutic approaches can be devised, thanks to current biotechnological innovations. The employment of MSCs may represent an alternative. Encouraging results regarding the degree of skeletal mineralization have been reported in infants with severe HPP treated with ex vivo expanded allogeneic MSCs [34].

## 6. MSCs and Cancer

MSCs have been associated with the tumor microenvironment as well. They are recruited to tumor sites and can be stimulated by TGF-β 1 to develop carcinoma-associated fibroblasts [35]. In breast cancer, tumor-initiating cells (TIC), also defined as cancer stem cells (CSC) with mesenchymal features, have been reported [36]. The transition of epithelial cells to mesenchymal cells (EMT) represents a physiological event during embryogenesis [37]. This process, however, which is characterized by the downregulation of E-cadherin, the production and secretion of matrix metalloproteases and the upregulation of mesenchymal markers, also occurs in several tumors such as in breast, ovarian and colon cancer [38]. Interestingly, it has been reported that the EMT induction of tumor cells is due to important stimulators of skeletal metastasis formation. In fact, among transcription factors involved in EMT, Snail1/2, Slug, Twist1 and Zeb1/2 have been reported [37]. Furthermore, the TGFβ and WNT pathways are known to induce oncogenic EMT and signaling pathways [39]. Recently, we reported that the osteogenic transcription factor RUNX2 may be considered a mesenchymal stem cell marker for cancer and that overexpression of this gene in solid tumors such as prostate, breast, pancreatic and lung cancer is associated with bone metastases [40]. Abnormal mesenchymal cell differentiation and overexpression of proto-oncogenes and downregulation of onco-suppressors are involved in the pathogenesis of osteosarcoma, a devastating bone tumor with a poor prognosis that affects children and adolescents. Mesenchymal stem cells or osteoblast cells have been identified as osteosarcoma-initiating cells and the epithelial to mesenchymal transition (EMT) has a pivotal role in this malignancy [41]. In particular, it has been reported that transcription factor mediators of EMT Twist, Snails and Zebs are involved in osteosarcoma pathogenesis and they have been considered targets for osteosarcoma treatment [41].

## 7. Clinical Applications of MSCs in Bone Regeneration and Repair

Many studies have documented the employment of autologous MSCs (either cultured or uncultured) in orthopedics to enable the repair of large bone defects [42,43].

Preclinical studies in osteoporosis animal models indicated the potential of autologous or allogeneic MSCs to engraft in recipients’ bones, improving bone mineral density and biomechanical stiffness [44]. The first human clinical trial to treat “classical” (i.e., collagen-related) OI with allogeneic bone marrow MSCs was performed by Horwitz and colleagues in 2002 [45]. Pre- and post-natal transplantation of allogeneic human fetal MSCs in two patients with OI was reported more recently [46].

Clinical trials regarding allogeneic bone marrow transplantation (BMT) in HPP patients have also been reported. Encouraging results were obtained recently in two patients with severe HPP by combining BMT and ex vivo expanded MSCs [34]. These and other clinical trials exploiting MSCs for the treatment of systemic bone diseases suggest that cell therapy is of likely clinical benefit but, unfortunately, the beneficial effects are transient. The exact dosage and routes of application remain to be optimized, and the fate of the transplanted cells and their mechanisms of action need to be better understood.

## 8. Concluding Remarks

MSCs play a fundamental role in skeletal tissue homeostasis. Dysfunctions in their osteogenic commitment, which is regulated by complex molecular pathways, may be the cause of various bone diseases. Inborn defects in genes coding for key regulatory factors of MSC osteogenic commitment result in rare skeletal genetic disorders. Disturbances in MSC osteogenic differentiation caused by environmental factors may also jeopardize their fate and cause systemic disorders such as osteoporosis and cancer.

The introduction of iPSC technology represents a reliable tool for modeling diseases and seeking disease mechanisms. In addition, the analysis of molecular pathways involved in MSC differentiation may lead to the identification of potential targets for a wide range of bone diseases. Finally, advances in the field of stem cells highlight the promising prospect of the therapeutic application of MSCs in bone diseases.
